# Characterization of the complete chloroplast genome of *Glycyrrhiza uralensis* (Leguminosae), a traditional Chinese medicine

**DOI:** 10.1080/23802359.2019.1666050

**Published:** 2019-09-18

**Authors:** Guolun Jia, Peng Li, Qiang Zhu, Li Peng

**Affiliations:** a Northwest University, Xi’an, China;; b The State Key Laboratory of Seeding Bioengineering, Ningxia Forestry Intitute, Yinchuan, China;; c Xi’an International University, Xi’an, China;; d Key Lab of Ministry of Education for Protection and Utilization of Special Biological Resources in Western China, School of Life Sciences, Ningxia University, Yinchuan, China

**Keywords:** *Glycyrrhiza uralensis*, Leguminosae, chloroplast genome, Illumina sequencing, phylogenetic analysis

## Abstract

*Glycyrrhiza uralensis* is a tradational Chinese medicine of Leguminosae, which contains many chemicals, such as glycyrrhizin, liquiritin glycyrol, melatonin (*N*-acetyl-5-methoxy tryptamine), glycyrrhizol A, glycyrrhizol B and four known isoflavonoids (5-O-methylglycryol, isoglycyrol, 6,8-diisoprenyl-5,7,4′-trihydroxy isoflavone, gancaonin).

Illumina paired-end reads data was used to assemble the complete chloroplast (cp) genome. About 15,445,866 raw Paired-End Reads and the length distribution in 127,702 bp (GC content accounts for 34.3%). Eight PCG genes and six tRNA genes possess a single intron, while ycf3 has a couple of introns. Based on the concatenated coding sequences of cp PCGs, the phylogenetic analysis showed that *Glycyrrhiza uralensis* and *Glycyrrhiza inflata* (*MH321931*) are closely related to each other within the family Leguminosae.

*Glycyrrhiza uralensis* is a traditional Chinese medicine of Leguminosae, which contains many chemicals, such as glycyrrhizin, liquiritin, glycyrol (Xie et al. 2019), melatonin (*N*-acetyl-5-methoxy tryptamine) (Afreen et al. 2006), glycyrrhizol A, glycyrrhizol B and four known isoflavonoids (5-O-methylglycryol, isoglycyrol, 6,8-diisoprenyl-5,7,4′-trihydroxy isoflavone, gancaonin) (He et al. 2006). Many reports shows that *G. uralensis* has multiple types of pharmaceutical efficacy, including multiple types of pharmaceutical efficacy (An et al. 2018). Glycyrrhiza polysaccharide (GCP) has inhibition effect on tumor growth (Zhang et al. 2018).

A pair of inverted repeats (IRs), separated by a large single-copy region (LSC) and a small single-copy region (SSC), these four parts, constitute a conserved structure of the complete chloroplast (cp) genome (Wolfe et al. 1992; Lee et al. 2007). This report will be very important for studying the phylogenetic relationships of *Folium Sennae* and Leguminosae.

The fresh leaves of *G. uralensis* were collected in the Ningxia Forestry Institute (38°28′N, 106°16′E; Ningxia, NW China) and deposited at Pharmacognosy laboratory in Northwest University(A voucher specimen: GU190515). The modified CTAB method was used to extract the genomic DNA (Doyle and Doyle 1987). We constructed a shotgun library with Illumina HiSeq X Ten Sequencing System (Illumina, San Diego, CA) following the manufacturer’s specification. The program MITObim v1.8 (https://github.com/chrishah/MITObim) was used to assemble cp genome (Hahn et al. 2013) and *Glycyrrhiza inflata* (*MH321931*) as the initial reference. The map of the complete cp genome was generated through the web-based tool OGDRaw v1.2 (http://ogdraw.mpimp-golm.mpg.de/) (Lohse et al. 2013) and the complete cp genome sequence has been submitted to GenBank (accession number *MN199032*).

The complete cp genome is a circular double-stranded DNA molecule, which with a typical quadripartite structure. We got 15,445,866 raw Paired-End Reads and the length distribution in 127,702 bp (GC content accounts for 34.3%).

The sequencing result encodes 109 complete genes, containing 75 protein-coding genes, 30 transfer RNA genes and 4 ribosomal RNA genes. In addition, 6 tRNA genes (*trnA-UGC, trnG-UCC, trnI-GAU, trnK-UUU, trnL-UAA* and *trnV-UAC*) harbor a single intron. *AtpF, ndhA, ndhB, rpl2, rpl16, rpoC1, petD* and *petB*, these 8 PCG genes possess a single intron, 66 PCG genes no intron, *ycf3* harbor two introns.

Based on the concatenated coding sequences of 20 chloroplastp PCGs for 9 plastid genomes from published species of Leguminosae, we constructed a neighbour-joining (NJ) phylogenetic tree (Figure 1) using MEGA7 with 1000 bootstrap replicates (Kumar et al. 2016) (http://www.megasoftware.net/) to further study the phylogenetic position of *Folium Sennae.* From the NJ phylogenetic tree analysis, we find that *G. uralensis* and *Glycyrrhiza inflata* (*MH321931*) are closely related to each other within the family Leguminosae (Figure 1).

**Figure 1.**
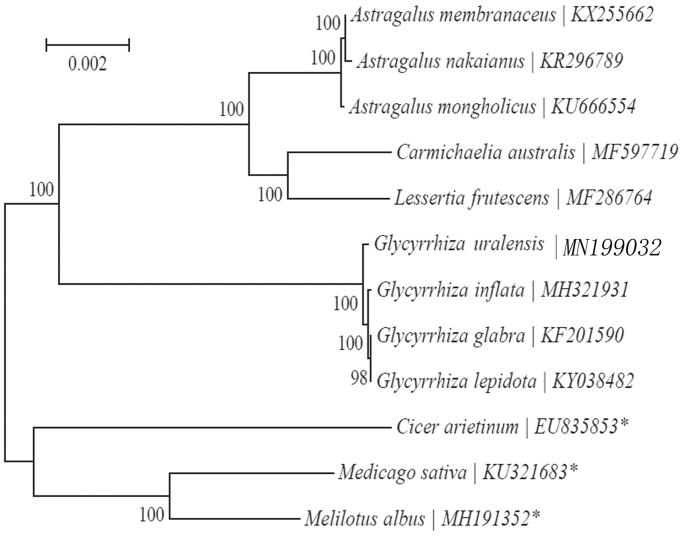
Maximum-likelihood (ML) tree of *G. uralensis* and its related relatives based on the complete chloroplast genome sequences.
